# RRT-CS: A free-collision planner for capsule-like SCORBOT by iterated learning

**DOI:** 10.1371/journal.pone.0323045

**Published:** 2025-05-19

**Authors:** Hung Nguyen, Thanh Phuong Nguyen, Song Hung Nguyen, Ha Quang Thinh Ngo

**Affiliations:** 1 HUTECH Institute of Engineering, HUTECH University, Ho Chi Minh, Vietnam; 2 Faculty of Mechanical Engineering, Ho Chi Minh City University of Technology (HCMUT), Ho Chi Minh, Vietnam; 3 Vietnam National University-Ho Chi Minh City (VNU-HCM), Ho Chi Minh, Vietnam; G H Raisoni College of Engineering and Management, Pune, INDIA

## Abstract

In this study, we present an enhanced Rapidly-exploring Random Trees (RRT) algorithm integrated with a visual servoing technique for recognizing unknown environments. The robotic platform utilized is the SCORBOT-ER-VII, which consists of five links, servo motors, gearboxes, and an end-effector. Several target objects are used to define the initial position, obstacles, and destination. To evaluate the effectiveness and robustness of our approach, we conducted both numerical simulations and hardware experiments across three test scenarios, ranging from obstacle-free environments to complex obstacle configurations. The results indicate that planning time increases proportionally with scenario complexity. The trajectory smoothing process accounts for less than 10% of the total processing time, while path shortening constitutes one-third, and RRT-based profile generation comprises the remaining two-thirds. These findings clearly demonstrate the efficiency of our approach in terms of computational time, making it well-suited for real-world applications.

## 1 Introduction

In the field of robotic control, autonomy is a key characteristic for exploring various environments [1,2], including unknown or foggy maps [3], dynamic obstacles, and emergency rescue scenarios [4]. As a fundamental research topic in autonomous robotics, motion planning has attracted significant attention. Most profile generation procedures require searching for multiple collision-free trajectories from the starting point to the destination [5–7]. In some cases, planning optimization also aims to minimize travel distance while satisfying additional constraints.

Probabilistic Roadmaps (PRM) and Rapidly-exploring Random Trees (RRT) are classified as sampling-based path planning algorithms, where a point is sampled within the working map and its nearest neighbor is explored from the existing tree. In the early stages of development, these methods prioritized rapid planning solutions over optimality. In [8], researchers highlighted that RRT is not asymptotically optimal and proposed Neural RRT*—a novel approach incorporating learning-based capabilities for potentially optimal path planning. This strategy is particularly suitable for practical applications such as autonomous driving and warehouse robotics. In [9], investigators leveraged convolutional neural networks to detect potential collisions while considering an optimal distance metric to estimate sample costs based on path curvature.

In this paper, our contributions are threefold: (i) introducing the robotic platform and related techniques, (ii) developing a motion planning scheme that integrates a robot control strategy with an advanced training model for a computer vision-based approach, and (iii) validating the proposed scheme on a real-world robotic system. The structure of this paper is as follows: Previous Works section reviews related works, followed by a description of the robotic platform, its parameters, the training model, and the camera setup in Preliminaries section. The Proposed Approach section presents the proposed approach, including the conceptual design, motion planner, and post-processing steps. Simulations and Experiments section discusses the experimental results, and Conclusions section concludes the paper with insights and directions for future research.

## 2 Previous works

Generally, RRT planners could be decomposed into various primitives while alterations or likenesses among them are visible [10–13]. Many scholars focused on the parameters and heuristics in sampling-based planners and their implementations in optimizing motion of hardware platform. The existing challenges in the sampling-based path planning are to cost large memory of computer, low speed of convergence, depend on nearest neighbor search and require post processing. On account of these troubles, several efforts for enhancing this motion planner have been made in recent years.

### 2.1 Sampling algorithm

Sampling process guides robot to extend the configuration space. Because it could spend a lot of time and cost in wide area and not all configurations are uniform, some algorithms have been investigated to overcome such as Voronoi graph [14,15], probabilistic roadmap method [16] or goal biasing [17].

### 2.2 Autonomous exploration

The ability of exploration becomes one of the key characteristics of robot in the complicated environment. In both visibility region [18] and multi-level region [19], adaptive sampling scheme is useful for autonomous robot even motion planner fails to discover.

### 2.3 Parameter metric

Most of the sampling-based strategies rely on metrics for their expandable search. Picking a proper metric is always hard for an operator so that it requires a good estimation and better understanding of this system [20,21]. Also, it can be used multiple times during the trajectory planning procedure.

### 2.4 Collision checking

One of the critical issues to evaluate the motion planner is to generate free-collision path. In some cases, it is classified into $C_{free}$ which provides free-collision path and $C_{obs}$ which contains the paths colliding with any obstacle. Collision checking would be called several times while robot is moving [22–24].

### 2.5 Robotic singularity

Conventionally, an industrial manipulator could be controlled in both joint space and Cartesian space. For joint-space driving commands, a reference set of joint positions should be provided. Later, it is translated or rotated each joint to the desired joint positions. For Cartesian-space motion commands, both the orientational and positioning pose of robotic gripper must be inserted. Then, motion planner would compute the inverse position and velocity kinematics for all robotic links. In this stage, a robot singularity in which its end-effector becomes blocked in certain direction, might arise. Consequently, there are several techniques to avoid such as vector field [25], geometric method [26] or genetic algorithm [27].

For further analysis, Table 1 summarizes the key limitations and its impact of the state-of-the-art researches in related topics. They are categorized in three groups which represent the separated applications. Then, in the same domain, they are classified owing to the specific techniques or definite design. Each of those researches is investigated to specify the key challenges and its effects in the theme of robotic control.

**Table 1:** List of the systematic analysis for the state-of-the-art researches.

Category	Issue	Key limitations	Impact
Path planning	Standard RRT [28]	Purely random sampling, no trajectory optimization	Results in suboptimal paths with excessive waypoints and sharp turns
	Enhanced RRT [29]	Asymptotically optimal but computationally expensive	Slow convergence, especially in high-dimensional spaces
	Bi-RRT [30]	Faster than RRT but lacks smoothing	Still prone to jerky motions, requires post-processing
	Learning-based RRT [31]	Requires large datasets and training time	Not adaptable to dynamic environments without retraining
Collision avoidance	Axis-Aligned Bounding Boxes (AABB) [32]	Overestimates obstacles, leading to overly conservative paths	Unnecessary detours
	Convex Hulls [33]	Fails to represent concave obstacles	Risk of false negatives (missed collisions)
	Point Clouds (Voxel-Based) [34]	Computationally expensive for real-time planning	Slow path updates
Visual solution for motion planning	Integrated interactive framework [35]	Be unable to provide continuous, reliable feedback for control	Becomes particularly problematic during large lateral displacements or in dynamic scenes where obstacles may occlude important features
	3D vision-based system [36]	Lack the necessary accuracy, robustness, and resolution in the existing vision systems, especially under variable lighting conditions or on uneven surfaces	The integration of advanced 3D vision technologies enhances the precision and reliability of damage recognition
	Safe and efficient navigation [37]	Suffer from issues such as random sampling inefficiencies, high memory demands, and limited adaptability to dynamic obstacles	It provides a comprehensive review of both global and local planning strategies
	Path planning in unstructured environment [38]	Struggle with adequately incorporating kinematic constraints and managing complex environmental dynamics, often resulting in inefficient or unsafe paths	The proposed improved kinematically constrained bi-directional RRT approach enhances planning efficiency and path quality. This advancement is expected to significantly benefit agricultural robotics by enabling safer and more reliable navigation in challenging, unstructured outdoor environments.

## 3 Preliminaries

To clarify our works, several components including hardware platform, model training and interactive objects, system configuration for both 5-DOF (Degree-Of-Freedom) robot and digital camera.

### 3.1 Robotic platform

**Fig 1:**
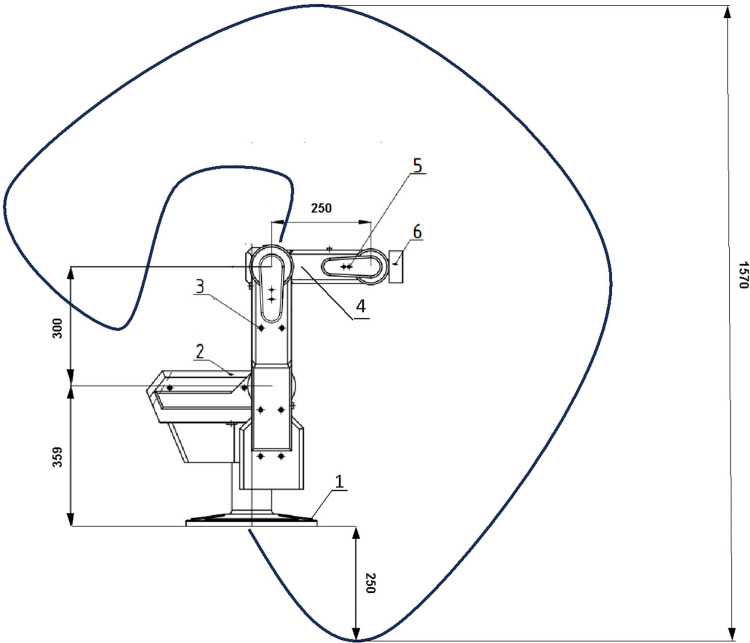
Workspace of the proposed robotic platform, (1) base platform, (2) joint 1, (3) socket head screw M5, (4) joint 3, (5) socket head screw M3 and (6) end-effector.

In this system, five DOFs robotic arm as Fig 1 is deployed to manipulate in front of objects. Its mechanical structure is vertical articulated hardware. There are five servo motors and harmonic drives, belt and pulley, and optical encoders to feedback signals. To pick an object, it is necessary to attach one robotic gripper at the end-effector. Optionally, two-finger gripper or three-finger gripper should be considered. However, to demonstrate the obstacle avoidance algorithm, robot arm without gripper is sometimes utilized.

### 3.2 Model training and target objects

Computer vision has attracted a lot of researches in various domains such as robotics, machining design, or information technology. It assists to capture, grasp, and analyze the highly understanding level of visual contents. To match with our purpose, object recognition, localization and segmentation are available to study. Currently, You Only Look Once (YOLO) [39] is very popular to detect an object with significantly high accuracy.

**Fig 2:**
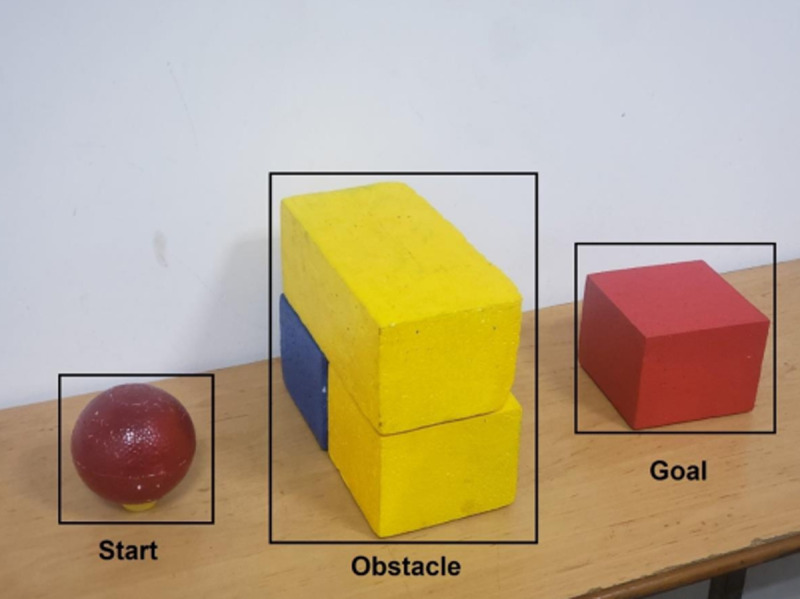
Target objects in our research.

**Fig 3:**
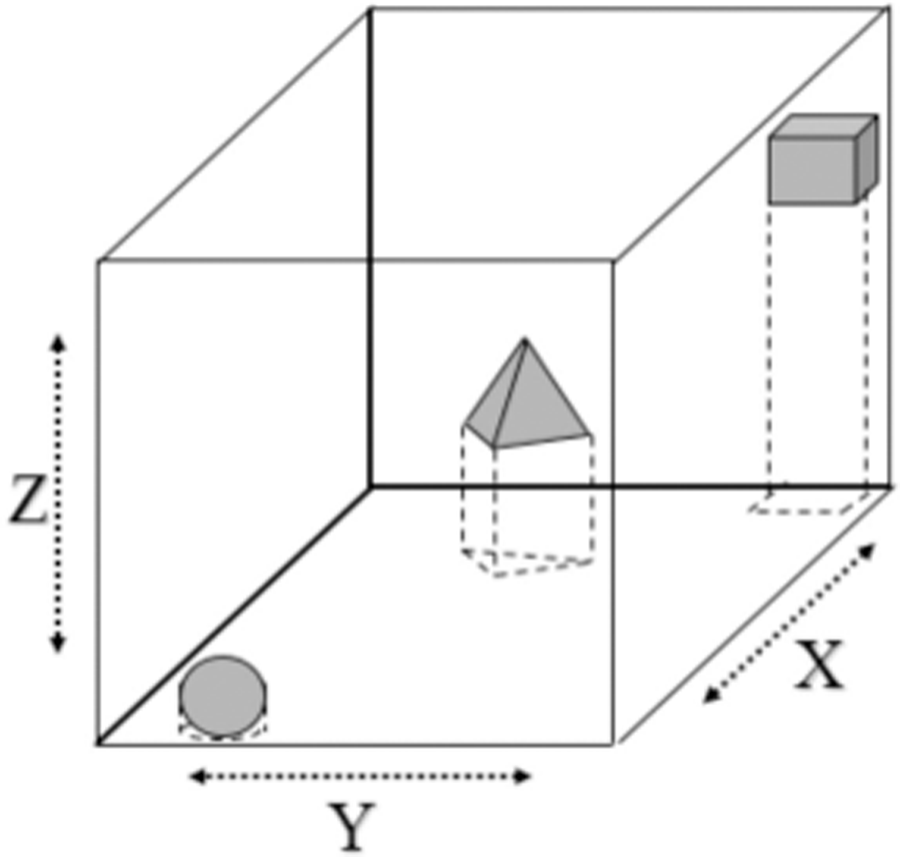
Illustration of scenario modeling.

To customize our detector, there are four steps such as data collection, data preparation, model training and model inference. In Fig 2, three kinds of objects are start, obstacle and goal. Dataset is collected from different views and shapes to provide precise detection. Robot must begin its task from start, avoid obstacle and toward to goal. Since these obstacles have rectangular shapes, the point-cloud coordinates of top-view corners of bounding box are A, B, C and D respectively. It is projected four corners down to table surface with depth *h* = *depth of ABCD* – *depth of table* as Fig 3.

**Fig 4:**
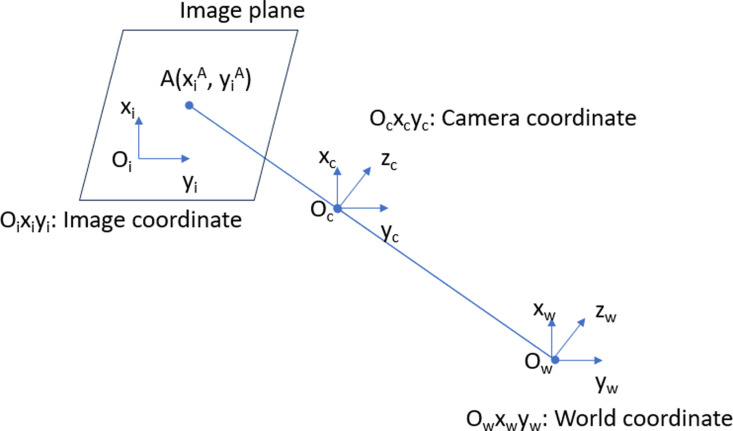
Relative constraints in multiple coordinates.


To obtain the 3D coordinate, the camera-inspired relative calibration is depicted as Fig 4. For more details, mathematical relations of these parameters are

$$s[\begin{array}{c} u\\ v\\ 1 \end{array}]=[\begin{array}{ccc} f_{x} & 0 & c_{x}\\ 0 & f_{y} & c_{y}\\ 0 & 0 & 1 \end{array}]\left[\begin{array}{ccc} r_{11} & r_{12} & r_{13}\\ r_{21} & r_{22} & r_{23}\\ r_{31} & r_{32} & r_{33} \end{array}\begin{array}{c} t_{1}\\ t_{2}\\ t_{3} \end{array}\right][\begin{array}{c} X\\ Y\\ \begin{array}{c} Z\\ 1 \end{array} \end{array}]$$ (1)

where

$\left[\begin{array}{ccc} f_{x} & 0 & c_{x}\\ 0 & f_{y} & c_{y}\\ 0 & 0 & 1 \end{array}\right]$: intrinsic matrix of digital camera, $f_{x},f_{y}$: focal length, $c_{x},c_{y}$: principal points

$\left[\begin{array}{c} u\\ v\\ 1 \end{array}\right]$: 2D image coordinate

$\left[\begin{array}{c} X\\ Y\\ \begin{array}{c} Z\\ 1 \end{array} \end{array}\right]$: 3D world coordinate

$\left[\begin{array}{ccc} r_{11} & r_{12} & r_{13}\\ r_{21} & r_{22} & r_{23}\\ r_{31} & r_{32} & r_{33} \end{array}\begin{array}{c} t_{1}\\ t_{2}\\ t_{3} \end{array}\right]$: extrinsic matrix of digital camera, $r_{11},r_{12},r_{13},r_{21},r_{22},r_{23},r_{31},r_{32},r_{33}$: camera rotation, $t_{1},t_{2},t_{3}$: camera translation

Using the Software Development Kit (SDK) of our camera, then intrinsic matrix of those parameters is measured as


$$\left[\begin{array}{ccc} f_{x} & 0 & c_{x}\\ 0 & f_{y} & c_{y}\\ 0 & 0 & 1 \end{array}]=[\begin{array}{ccc} 349.8 & 0 & 338.12\\ 0 & 349.8 & 190.3415\\ 0 & 0 & 1 \end{array}\right]$$ (2)

Later, relative transformations should be identified to convert the real-world coordinate to image coordinate on a Charge Coupled Device (CCD) sensor of camera. We assume that X, Y, Z are the actual coordinates of the object (mm) relative to the camera in space and x, y are the coordinates of the pixel (mm) on the CCD optical sensor. In Fig 3, we apply Thales theorem


$$\frac{Y}{Z}=\frac{y}{f_{y}},\frac{X}{Z}=\frac{x}{f_{x}}\to x=\frac{f_{x}X}{Z},y=\frac{f_{y}Y}{Z}$$ (3)

We have the conversion matrix of image coordinates on CCD and real coordinates as follows


$$\left[\begin{array}{c} \tilde{x}\\ \tilde{y}\\ \tilde{z} \end{array}]=T[\begin{array}{c} \begin{array}{c} X\\ Y \end{array}\\ \begin{array}{c} Z\\ 1 \end{array} \end{array}\right]$$ (4)

In which matrix: $T=[\begin{array}{cc} \begin{array}{cc} \begin{array}{c} f_{x}\\ 0\\ 0 \end{array} & \begin{array}{c} 0\\ f_{y}\\ 0 \end{array} \end{array} & \begin{array}{cc} \begin{array}{c} 0\\ 0\\ 1 \end{array} & \begin{array}{c} 0\\ 0\\ 0 \end{array} \end{array} \end{array}]$

Regularly, the image coordinates would be moved to the left corner of the image and considered as coordinates (0,0). The mm coordinates are converted to pixel coordinates.


$$x=\left(u-u_{0}\right)\times s_{x}\to u=\frac{x}{s_{x}}+u_{0}$$ (5)

$$y=\left(v-v_{0}\right)\times s_{y}\to v=\frac{y}{s_{y}}+v_{0}$$ (6)

where,

$u_{0},v_{0}$ are the pinhole center coordinate

$s_{x},s_{y}$ are the length of each pixel in mm in the x and y direction

The formula to convert mm coordinates on the frame to pixel coordinates is as follows


$$x=\left(u-u_{0}\right)\times s_{x}\to u=\frac{x}{s_{x}}+u_{0}=\frac{f_{x}X}{{Zs}_{x}}+u_{0}$$ (7)

$$y=\left(v-v_{0}\right)\times s_{y}\to v=\frac{y}{s_{y}}+v_{0}=\frac{f_{y}Y}{{Zs}_{y}}+v_{0}$$ (8)

Hence,


$$p^{\prime }=[\begin{array}{c} su\\ sv\\ s \end{array}]=[\begin{array}{ccc} \begin{array}{c} \frac{f_{x}}{s_{x}}\\ 0\\ 0 \end{array} & \begin{array}{c} 0\\ \frac{f_{y}}{s_{y}}\\ 0 \end{array} & \begin{array}{c} u_{0}\\ v_{0}\\ 1 \end{array} \end{array}]=K[\begin{array}{c} X\\ Y\\ Z \end{array}],s=Z$$ (9)

$$p=[\begin{array}{c} u\\ v\\ 1 \end{array}]=\frac{p^{\prime }}{s}$$ (10)

K: Internal parameter matrix of the camera

We suppose that this camera has coordinates relative to a different origin system. As a result, we have a general formula that transforms from the system


$$\left[\begin{array}{c} su\\ sv\\ s \end{array}]=K\times M\times [\begin{array}{c} \begin{array}{c} X_{w}\\ Y_{w} \end{array}\\ \begin{array}{c} Z_{w}\\ 1 \end{array} \end{array}\right]$$ (11)

where,

M: external parameter matrix used to convert object coordinates from global coordinates to camera coordinates. In order to convert a pixel coordinate to real coordinates, we proceed as following


$$Zm_{p}=KM_{c}=K\left(RM_{W}+T\right)=>Zm_{p}=K\left(RM_{W}+T\right)=>M_{W}=R^{-1}(K^{-1}Zm_{p}-T)$$ (12)

$m_{p}=[\begin{array}{c} u\\ v\\ 1 \end{array}]$: pixel coordinate and $M_{W}=[\begin{array}{c} X\\ Y\\ Z \end{array}]$: global coordinates

Camera distance to the workspace Z=350 (mm). We have the internal matrix of the camera $K=[\begin{array}{ccc} \begin{array}{c} \frac{349.3}{350}\\ 0\\ 0 \end{array} & \begin{array}{c} 0\\ \frac{347.4}{350}\\ 0 \end{array} & \begin{array}{c} 338\\ 198\\ 1 \end{array} \end{array}]$. In order to convert a point from one coordinate system to another (in this case, change from camera coordinate system to robot coordinate system), we need to find the transfer matrix M


$$\begin{array}{ccc} P_{R} & = & M_{trans}P_{C}\\ ↔[\begin{array}{c} \begin{array}{c} X\\ Y \end{array}\\ \begin{array}{c} Z\\ 1 \end{array} \end{array}] & = & \left[\begin{array}{cc} \begin{array}{cc} R_{11} & R_{12}\\ R_{21} & R_{22} \end{array} & \begin{array}{cc} R_{13} & T_{X}\\ R_{23} & T_{Y} \end{array}\\ \begin{array}{cc} R_{31} & R_{32}\\ 0 & 0 \end{array} & \begin{array}{cc} R_{33} & T_{Z}\\ 0 & 1 \end{array} \end{array}][\begin{array}{c} \begin{array}{c} x\\ y \end{array}\\ \begin{array}{c} z\\ 1 \end{array} \end{array}\right] \end{array}$$ (13)

where,

P_R_: The known coordinates in advance of the robot coordinate system

P_C_: The known coordinates in advance of the camera coordinate system

M_trans_: transformation matrix

**Fig 5:**
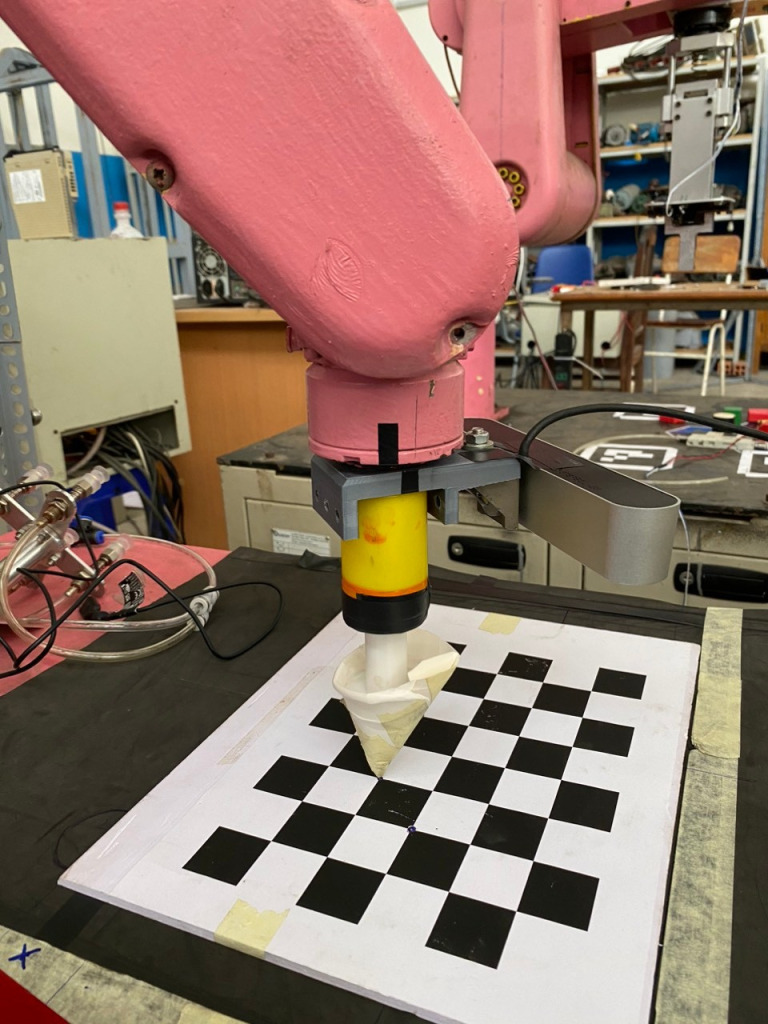
Demonstration of the first step for points taking process.

**Fig 6:**
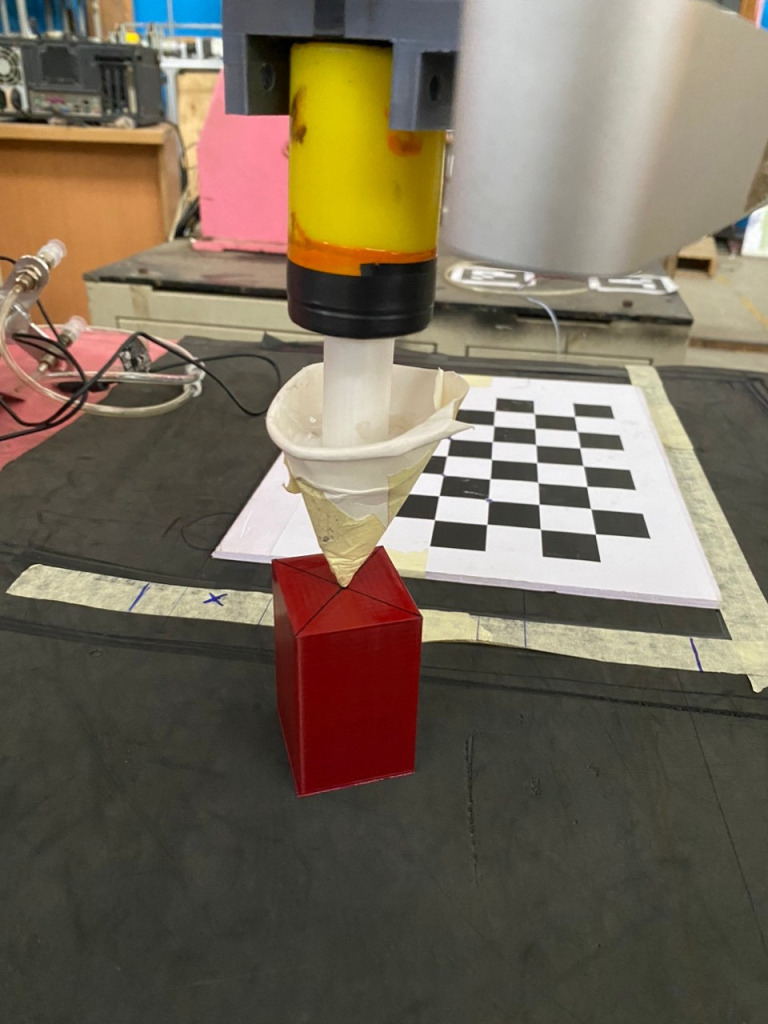
Demonstration of the second step for points taking process.

To determine the M matrix according to above equations, it is necessary to find any four points in local coordinates by moving robotic arm to four different points in the workspace as Fig 5. After that, its values in pixels are recorded by using a camera as Fig 6.

For avoiding the appearance of outliers, four selected points as Table 2 must have at least one point which is not on the same plane as the remaining points (specifically z much be different).

**Table 2:** Results of measurements in relative parameters.

*Robot Coordinate* *(X, Y, Z)*	(360,150,183)	(357,207,183)	(387,120,183)	(340, −65,240)
*Camera Coordinate* *(x, y, z)*	(178,185,465)	(134,186,465)	(200,163,465)	(355,200,400)

At that point, applying these parameters to the formular, we get:


$$M_{trans}=[\begin{array}{cccc} 0 & -1 & 0 & 504\\ -1 & 0 & 0 & 460\\ 0 & 0 & -1 & 591\\ 0 & 0 & 0 & 1 \end{array}]$$ (13)

Therefore, we obtain relative parameters during our calibration as Table 3.

**Table 3:** Results of relative parameters in the process of camera calibration.

Experiment coordinate x	Experiment coordinate y	Robot x coordinate	Robot y coordinate	Displacement x	Displacement y
329	98	325	93	1.24%	5.38%
355	117	345	112	2.9%	4.47%
290	163	304	165	3.34%	1.22%
361	226	353	225	0.84%	0.45%
301	163	310	165	2.91%	1.22%
423	60	435	55	0.48%	9.1%
411	117	421	110	2.15%	6.37%
400	107	415	100	3.62%	7%
431	227	426	220	1.18%	3.19%
250	97	246	100	1.63%	3%
272	111	264	115	0.75%	3.48%
260	218	248	225	1.97%	3.12%
305	241	295	245	3.39%	1.64%
250	97	239	100	1.24%	5.38%
**Overall**				2.04%	3.82%

### 3.3 Forward kinematic

**Fig 7:**
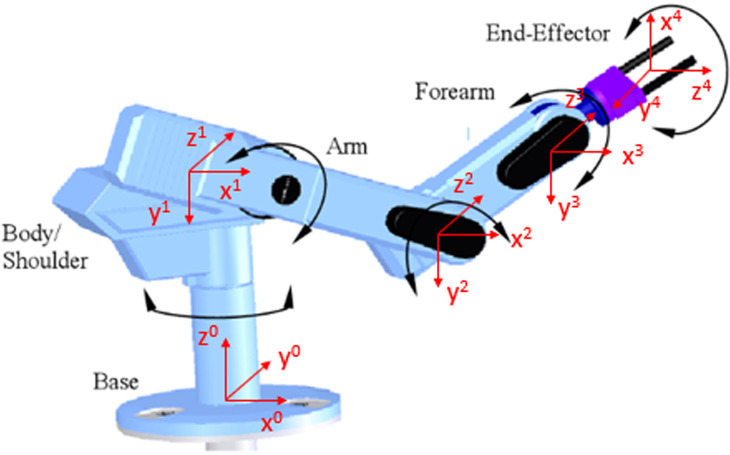
Modeling of joint-coordinate for our manipulator.


Systematically, forward kinematic challenge could be solved by defining Denavit-Hartenberg (D-H) parameters as Table 4. It is a method to find homogenous transformation matrices among serial links of robot arm as [40]. $d_{tool}$ is the distance from the center of 4^th^ joint to the center of our gripper as Fig 7. Owing to this design, the homogenous transformation matrix $\backslash {(}_{i}^{i-1}T$: ca is generally well-defined as below

**Table 4:** D-H table for robotic parameters.

Joint	$d_{i}(mm)$	$a_{i}(mm)$	$\alpha_{i}(rad)$	$\theta_{i}(rad)$
1	358,5	50	$-\frac{\pi }{2}$	$\theta_{1}$
*2*	0	300	0	$\theta_{2}$
*3*	0	250	0	$\theta_{3}$
4	0	0	$-\frac{\pi }{2}$	$\theta_{4}$
*5*	$d_{S}=d_{tool}$	0	0	$\theta_{5}$

$$\backslash {(}_{i}^{i-1}T=\left[\begin{array}{ccc} \cos\theta_{i} & -sin\theta_{i}\cos\alpha_{i} & \sin\theta_{i}\sin\alpha_{i}\\ \sin\theta_{i} & \cos\theta_{i}\cos\alpha_{i} & -cos\theta_{i}\sin\alpha_{i}\\ \begin{array}{c} 0\\ 0 \end{array} & \begin{array}{c} \sin\alpha_{i}\\ 0 \end{array} & \begin{array}{c} \cos\alpha_{i}\\ 0 \end{array} \end{array}\begin{array}{c} a_{i}\cos\theta_{i}\\ a_{i}\sin\theta_{i}\\ \begin{array}{c} d_{i}\\ 1 \end{array} \end{array}\right]$$ (14)

$$\backslash {(}_{1}^{0}T=\left[\begin{array}{ccc} \cos\theta_{1} & 0 & -sin\theta_{1}\\ \sin\theta_{1} & 0 & \cos\theta_{1}\\ \begin{array}{c} 0\\ 0 \end{array} & \begin{array}{c} -1\\ 0 \end{array} & \begin{array}{c} 0\\ 0 \end{array} \end{array}\begin{array}{c} 50cos\theta_{1}\\ 50sin\theta_{1}\\ \begin{array}{c} 358,5\\ 1 \end{array} \end{array}\right]$$ (15)

$$\backslash {(}_{2}^{1}T=\left[\begin{array}{ccc} \cos\theta_{2} & -sin\theta_{2} & 0\\ \sin\theta_{2} & \cos\theta_{2} & 0\\ \begin{array}{c} 0\\ 0 \end{array} & \begin{array}{c} 0\\ 0 \end{array} & \begin{array}{c} 1\\ 0 \end{array} \end{array}\begin{array}{c} 300cos\theta_{2}\\ 300sin\theta_{2}\\ \begin{array}{c} 0\\ 1 \end{array} \end{array}\right]$$ (16)

$$\backslash {(}_{3}^{2}T=\left[\begin{array}{ccc} \cos\theta_{3} & -sin\theta_{3} & 0\\ \sin\theta_{3} & \cos\theta_{3} & 0\\ \begin{array}{c} 0\\ 0 \end{array} & \begin{array}{c} 0\\ 0 \end{array} & \begin{array}{c} 1\\ 0 \end{array} \end{array}\begin{array}{c} 250cos\theta_{3}\\ 250sin\theta_{3}\\ \begin{array}{c} 0\\ 1 \end{array} \end{array}\right]$$ (17)

$$\backslash {(}_{4}^{3}T=\left[\begin{array}{ccc} \cos\theta_{4} & 0 & -sin\theta_{4}\\ \sin\theta_{4} & 0 & \cos\theta_{4}\\ \begin{array}{c} 0\\ 0 \end{array} & \begin{array}{c} -1\\ 0 \end{array} & \begin{array}{c} 0\\ 0 \end{array} \end{array}\begin{array}{c} 0\\ 0\\ \begin{array}{c} 0\\ 1 \end{array} \end{array}\right]$$ (18)

$$\backslash {(}_{5}^{4}T=\left[\begin{array}{ccc} \cos\theta_{5} & -sin\theta_{5} & 0\\ \sin\theta_{5} & \cos\theta_{5} & 0\\ \begin{array}{c} 0\\ 0 \end{array} & \begin{array}{c} 0\\ 0 \end{array} & \begin{array}{c} 1\\ 0 \end{array} \end{array}\begin{array}{c} 0\\ 0\\ \begin{array}{c} d_{tool}\\ 1 \end{array} \end{array}\right]$$ (19)

Thus, the tool coordinate is computed as


$$\backslash {(}_{5}^{0}T{=}_{1}^{0}T_{2}^{1}T_{3}^{2}T_{4}^{3}T_{5}^{4}T=\left[\begin{array}{ccc} R_{11} & R_{12} & R_{13}\\ R_{21} & R_{22} & R_{23}\\ \begin{array}{c} R_{31}\\ 0 \end{array} & \begin{array}{c} R_{32}\\ 0 \end{array} & \begin{array}{c} R_{33}\\ 0 \end{array} \end{array}\begin{array}{c} P_{x}\\ P_{y}\\ \begin{array}{c} P_{z}\\ 1 \end{array} \end{array}\right]$$ (20)

where


$$R_{11}=cos\left(\theta_{4}+\theta_{3}+\theta_{2}\right)\cos\left(\theta_{1}\right)\cos\left(\theta_{5}\right)+sin\left(\theta_{1}\right)\sin\left(\theta_{5}\right)$$

$$R_{12}=-cos\left(\theta_{4}+\theta_{3}+\theta_{2}\right)\cos\left(\theta_{1}\right)\cos\left(\theta_{1}\right)\cos\left(\theta_{5}\right)+sin\left(\theta_{1}\right)\cos\left(\theta_{5}\right)$$

$$R_{13}=-sin\left(\theta_{4}+\theta_{3}+\theta_{2}\right)\cos\left(\theta_{1}\right)$$

$$R_{21}=cos\left(\theta_{4}+\theta_{3}+\theta_{2}\right)\sin\left(\theta_{1}\right)\cos\left(\theta_{5}\right)-cos\left(\theta_{1}\right)\sin\left(\theta_{5}\right)$$

$$R_{22}=-cos\left(\theta_{4}+\theta_{3}+\theta_{2}\right)\sin\left(\theta_{1}\right)\sin\left(\theta_{5}\right)-cos\left(\theta_{1}\right)\cos\left(\theta_{5}\right)$$

$$R_{23}=-sin\left(\theta_{4}+\theta_{3}+\theta_{2}\right)\sin\left(\theta_{1}\right)$$

$$R_{31}=-sin\left(\theta_{4}+\theta_{3}+\theta_{2}\right)\cos\left(\theta_{5}\right)$$

$$R_{32}=sin\left(\theta_{4}+\theta_{3}+\theta_{2}\right)\sin\left(\theta_{5}\right)$$

$$R_{33}=-cos\left(\theta_{4}+\theta_{3}+\theta_{2}\right)$$

$$P_{x}=cos\left(\theta_{1}\right)\left[250cos\left(\theta_{3}+\theta_{2}\right)+300cos\left(\theta_{2}\right)-d_{tool}\sin\left(\theta_{4}+\theta_{3}+\theta_{2}\right)+50\right]$$

$$P_{y}=sin\left(\theta_{1}\right)\left[250cos\left(\theta_{3}+\theta_{2}\right)+300cos\left(\theta_{2}\right)-d_{tool}\sin\left(\theta_{4}+\theta_{3}+\theta_{2}\right)+50\right]$$

$$P_{z}=-300sin\left(\theta_{2}\right)-250sin\left(\theta_{3}+\theta_{2}\right)-d_{tool}\cos\left(\theta_{4}+\theta_{3}+\theta_{2}\right)+358,5$$

### 3.4 Inverse kinematic

Target solution of inverse kinematic is to find the potential configurations of the set of joint angles *θ* of this robotic platform when the end-effector towards to destination


$$\theta =\left[\begin{array}{ccc} \theta_{1} & \theta_{2} & \theta_{3} \end{array}\begin{array}{cc} \theta_{4} & \theta_{5} \end{array}\right]$$ (21)

In this stage, all values of homogenous transformation matrix in equation (8) are given. By solving trigonometric equation, we have


$$\frac{P_{y}}{P_{x}}=\frac{\sin\theta_{1}}{\cos\theta_{1}}=tan\theta_{1}$$

$$\to \theta_{1}=\tan^{-1}\frac{P_{y}}{P_{x}}$$ (22)

$$\frac{R_{32}}{R_{31}}=-\frac{\sin\theta_{5}}{\cos\theta_{5}}=-tan\theta_{5}$$

$$\to \theta_{5}=-\tan^{-1}\frac{R_{32}}{R_{31}}$$ (23)

$${And\theta }_{2}+\theta_{3}+\theta_{4}=\cos^{-1}R_{33}$$ (24)

$$P_{x}\cos\theta_{1}+P_{y}\sin\theta_{1}=250cos\left(\theta_{2}+\theta_{3}\right)+300cos\theta_{2}-d_{tool}\sin\left(\theta_{2}+\theta_{3}+\theta_{4}\right)+50$$ (25)

Hence,


$$\left\{ \begin{array}{c} 250cos\left(\theta_{2}+\theta_{3}\right)+300cos\theta_{2}=P_{x}\cos\theta_{1}+P_{y}\sin\theta_{1}\\ \begin{array}{c} +d_{tool}\sin\left(\theta_{2}+\theta_{3}+\theta_{4}\right)-50\\ \begin{array}{c} 250sin\left(\theta_{2}+\theta_{3}\right)+300sin\theta_{2}=-P_{z}+358,5\\ -d_{tool}\sin\left(\theta_{2}+\theta_{3}+\theta_{4}\right) \end{array} \end{array} \end{array}\right.$$ (26)

Let


$$A_{e}=P_{x}\cos\theta_{1}+P_{y}\sin\theta_{1}+d_{tool}\sin\left(\theta_{2}+\theta_{3}+\theta_{4}\right)-50$$ (27)

$$B_{e}=-P_{z}+358,5-d_{tool}\sin\left(\theta_{2}+\theta_{3}+\theta_{4}\right)$$ (28)

Then, equation (14) becomes


$$\left\{ \begin{array}{c} 300cos\theta_{2}-A_{e}=-250cos\left(\theta_{2}+\theta_{3}\right)\\ 300sin\theta_{2}-B_{e}=-250sin\left(\theta_{2}+\theta_{3}\right) \end{array}\right.$$ (29)

$$\to \frac{A_{e}}{\sqrt{A_{e}^{2}+B_{e}^{2}}}{cos\theta }_{2}+\frac{B_{e}}{\sqrt{A_{e}^{2}+B_{e}^{2}}}{sin\theta }_{2}=\frac{A_{e}^{2}+B_{e}^{2}-27500}{600}$$ (30)

We get


$$\sin\theta_{e}=\frac{A_{e}}{\sqrt{A_{e}^{2}+B_{e}^{2}}}$$ (31)

$$\cos\theta_{e}=\frac{B_{e}}{\sqrt{A_{e}^{2}+B_{e}^{2}}}$$ (32)

$$\to \theta_{2}=\sin^{-1}\left(\frac{A_{e}^{2}+B_{e}^{2}-27500}{600\sqrt{A_{e}^{2}+B_{e}^{2}}}\right)-\theta_{e}$$ (33)

$$\to \theta_{3}=\cos^{-1}\left(\frac{A_{e}-300cos\theta_{2}}{250}\right)-\theta_{2}$$ (34)

In case that there are multiple inverse kinematic results, unique solution must be voted according to these constraints on the joint limit angle.

### 3.5 Camera installation

The set-up configuration of vision system is eye2hand method. It consists of two steel bars, metal load and base. Since it looks like console beam, there is a counter-load in the other end of bar while digital camera is hung in one end. This configuration ensures global coverage, stable image.

To identify the dimension of camera standee, some geometric constraints should be considered. With vertical FOV (Field of View), it must cover the working space of robot such (z×x×y):(400×800×800)(mm). The potential dimension could be estimated as


$$h\geq \frac{800}{2}\times cotan\left(\frac{60^{o}}{2}\right)+400\approx 1093(mm)$$ (35)

$$x\geq h\times tan\left(\frac{60^{o}}{2}\right)+100\approx 740(mm)$$ (36)

In addition, force/moment is analyzed to balance without tipping over. The mass of counter-load must satisfy below condition as


$$\sum iM_{i}^{B}=0$$ (37)

$$↔F_{b}\times L_{2}+F_{c}\times L_{3}-F_{w}\times L_{1}=0$$

$$↔m_{c}=\frac{F_{w}\times L_{1}-F_{b}\times L_{2}}{g\times L_{3}}$$

where

$F_{c}$: force by weight of counter-load

$F_{b}$: force by weight of steel bar. It locates at the center of bar

$F_{w}$: force by weight of digital camera

## 4 The proposed approach

**Fig 8:**
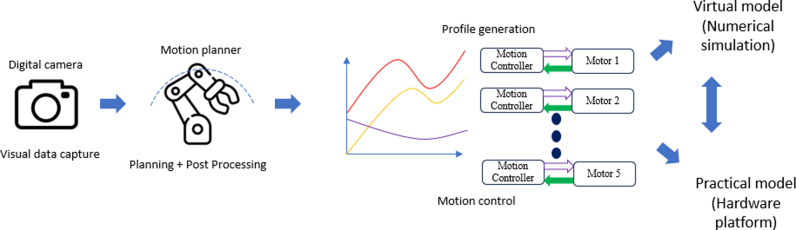
Overall scheme for our development.

In this investigation, the proposed design as Fig 8 comprises four components such workspace data capture, motion planner, profile generation and robotic platform. Objects in the working space are captured by a powerful camera and sent to host computer. Later, data is extracted to achieve necessary spatial information of start, goal, and obstacles. Path planner sketches out the candidate traveling routes and elect the shortening collision-free one. Then, motion data is exchanged and interpolated using inverse kinematic procedure. Additionally, for each link, various profiles are generated to ensure the smooth motion in time-space domain. Our design is firstly validated to obtain reference path in Matlab software, consequently command data is input to drive mechanical robot. Whole system could be supervised by an operator via digital camera.

### 4.1 General concept

Main program which is embedded into microprocessor, involves three fundamental steps. Initially, the surrounding context that could be captured by digital camera, plays as input of sub-program. Secondly, motion planner is triggered to elect the shortening collision-free route in RRT path planning sub-program. Thirdly, driving command is performed in the level of motion controller.

From the visual data, it is fed to YOLO algorithm for object detection involved in path planning. It returns the type of objects, 2D bounding boxes and its centroid pixel coordinate of each box. Moreover, software development kit (SDK) [41] of camera is utilized to get 3D coordinates of multiple point-clouds. Subsequently, it is multiplied with extrinsic matrix to transform these 3D coordinates to the robot basement. According to the type of detected objects as above, the occupied volume in workspace could be determined via its shape and centroid coordinate.

To lessen the heavy computations, robotic platform requires to simplify its links into a simpler geometric model such as ellipsoids [42], cylinders [43], and spheres [44]. In this work, our concept is to deploy a model-based capsule for stimulating the robotic links. This approach is not only well fitted in the shape of robotic arm but also deliver the efficient computations. Due to this design, each solid segment represents the skeleton of robotic manipulator. Similarly, an object or obstacle is also modeled as the shaded capsules and the distance among capsules of the closest pair is very sensitive to avoid collision. We assume that robotic platform at the state $q\in {\mathbb{R}}^{N}$ occupies its workspace in the Cartesian space $C(q)\in {\mathbb{R}}^{3}$. Then, an object with its state $x_{0}$ also occupies an area in the environment $\Theta (x_{0})\in {\mathbb{R}}^{3}$. It is supposed that the Euclidean distance vector from position of robot ${𝒫}_{r0}$ to position of object ${𝒫}_{ob}$: ${\mathbb{R}}^{3}\times {\mathbb{R}}^{3}\to {\mathbb{R}}^{3}$, consequently the norm of relative distance vector between robot and object is identified as


$$∥d_{r0}(q,x_{0})∥=min∥d_{i}({𝒫}_{r0},{𝒫}_{{ob}^{i}})∥{𝒫}_{r0}\in C(q),{𝒫}_{{ob}^{i}}\in \Theta (x_{0})$$ (38)

**Fig 9:**
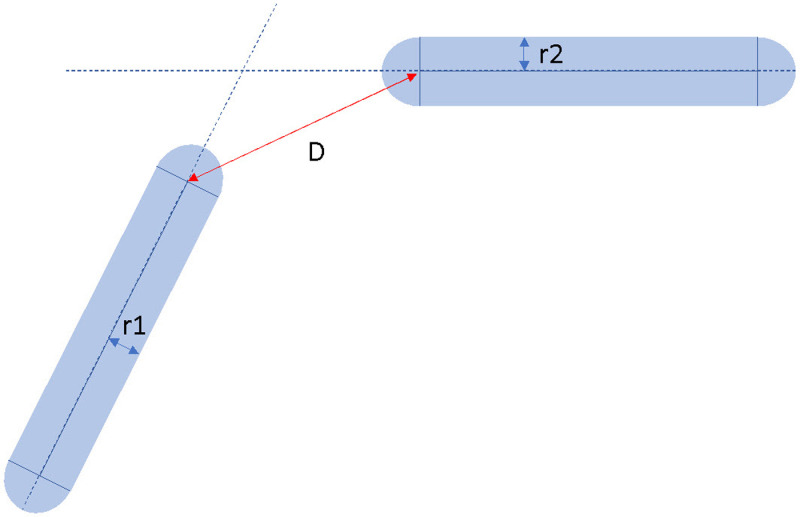
Estimation of distance between two capsules.

**Fig 10:**
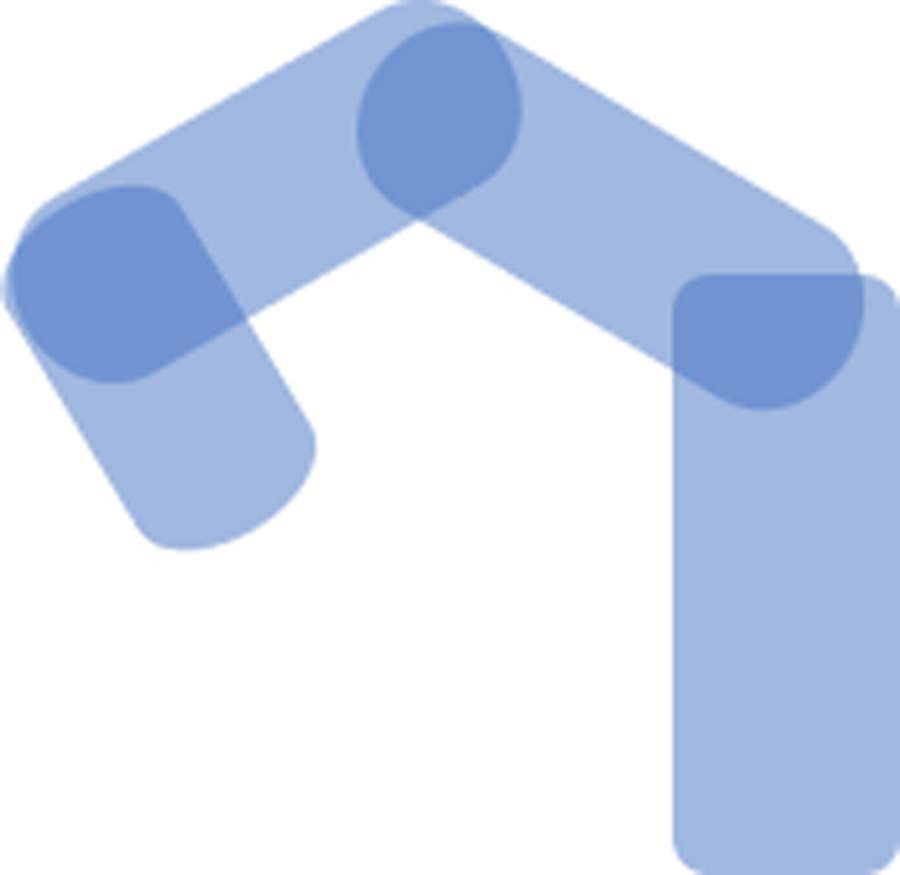
Illustration of capsule-like robot in our research.

In Fig 9, it is denoted that robotic links are represented as geometric model with different dimensions, for instance long or short capsule, indicating hardware consumption in workplace. Thus, our concept of capsule-like SCORBOT is proposed as Fig 10. This capsule shape comprises a cylinder with two semi-spherical ends, has the same width as its physical size of link. Henceforth, value $d_{ro}$ becomes distance between two axes. Our robotic platform is modeled as three bounding volumes since they have the highest probability of collision when robot is moving.

### 4.2 Path planner

**Fig 11:**
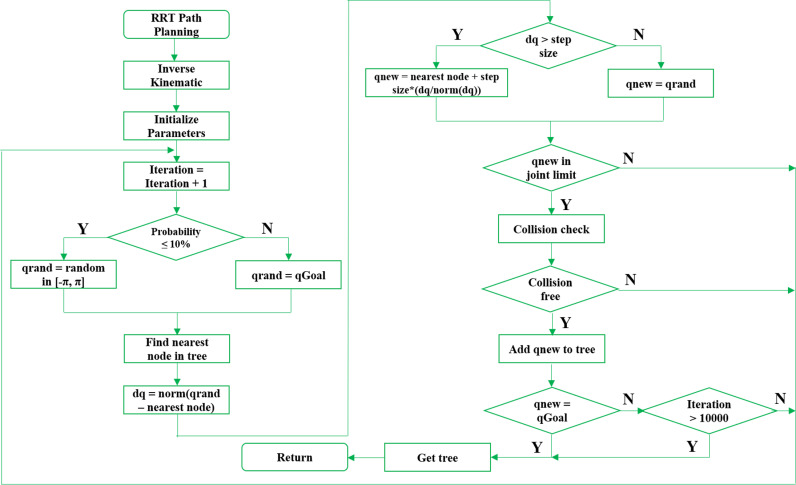
The proposed path planning scheme for SCORBOT-ER-VII.

The core idea of our path planning is to randomly generate a tree in the searching space, and maintain the Euclidian distance among nodes less than or equal to step size. If a new node might be in collision, it should be ignored and get another new node. This scheme is terminated if the goal is reached, and then the path planning is obtained. Due to our design, the proposed path planner is illustrated as Fig 11. From the start and goal, the step of inverse kinematic returns a random configuration $q_{rand}=\left[q_{1},q_{2},q_{3},q_{4},q_{5}\right]$. Goal bias factor α is defined as upper limit to improve the planning convergence in faster and higher success rate. If the random probability is equal or less than its value, $q_{rand}$ would be the goal configuration. Otherwise, it would be random configuration in range of −π and π. In fact, this factor α should be experimentally tuned based on a trade-off between exploration and convergence. A higher α speeds up convergence but increases the risk of local minima, while a lower α ensures better exploration but slows convergence. In our best knowledge, the proposed algorithm achieves a 30% faster convergence rate in simple scenarios when α equals to 0.3. In more complicated environments, our algorithm ensures higher success rates if the value of this factor α is 0.6.

After obtaining $q_{rand}$, the nearest configuration would be found and the Euclidian distance $d_{ro}$ between $q_{rand}$ and nearest one should be computed. If $d_{ro}$ is greater than step size, $q_{new}$ becomes the nearest one plus $q_{rand}$ configuration with step size length. Or else, new configuration would be $q_{rand}$ immediately. After that, this configuration would be checked for joint limit and collision conditions.

### 4.3 Path post processing

**Fig 12:**
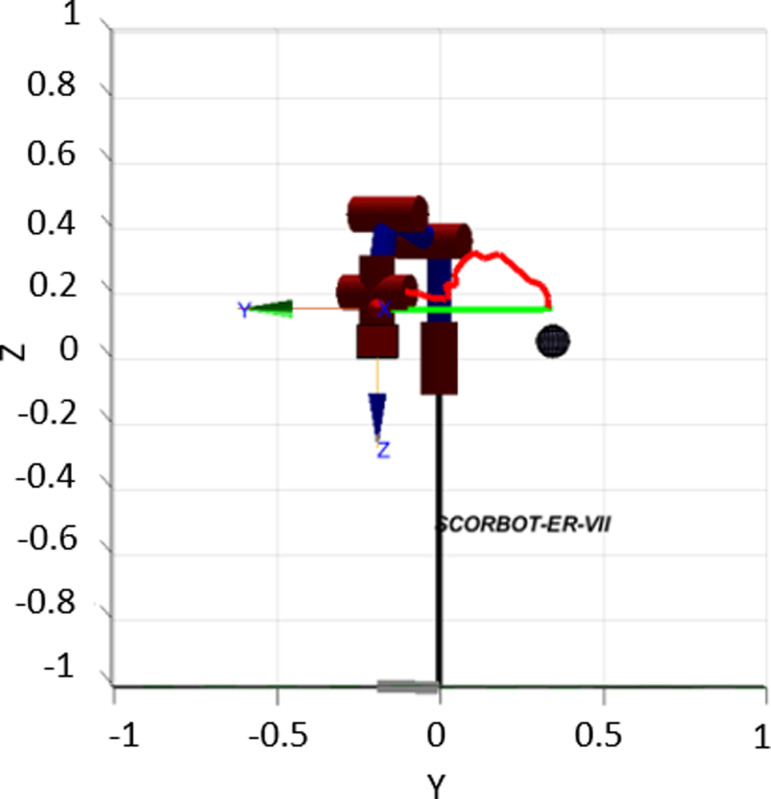
Description of traditional RRT path (red color) and post processed RRT path (green color).

In fact, path planning by traditional RRT is not optimal since it has many redundant nodes and sharp turning point as Fig 12. This path is not proper for driving manipulator in term of traveling distance and smoothness. The modified RRT path planning scheme must satisfy two requirements such that reducing path nodes are free collision and sudden change should be avoided for reliably mechanical system and safely human interaction.

**Fig 13:**
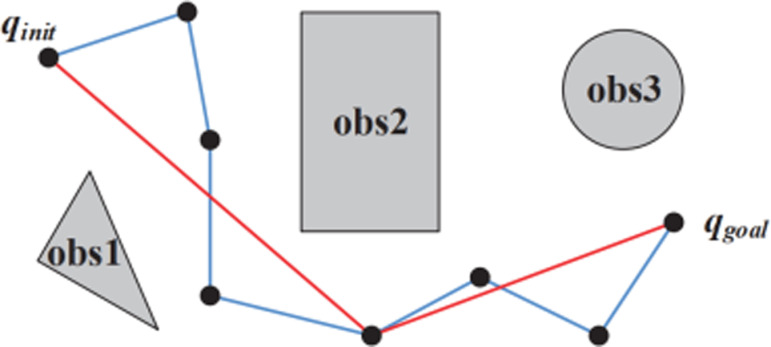
Description of traditional RRT path (green color) and path shortening technique (red color).

Our motivation to shorten traveling path is to assign a temporary configuration $q_{temp}$ as q[0] at start, and then connect q[0] with q[i],i=1,2,…N checked for potential collision sequentially. If there is no collision, the validation for next configuration should be kept without changing $q_{temp}$. Else, changing $q_{temp}$ to previous configuration q[i−1], add $q_{temp}$ to the reduced tree, and do the same collision-checking procedure for the next configuration q[i+1]. It would be ended when the checking process reaches to the last configuration as Fig 13.

**Fig 14:**
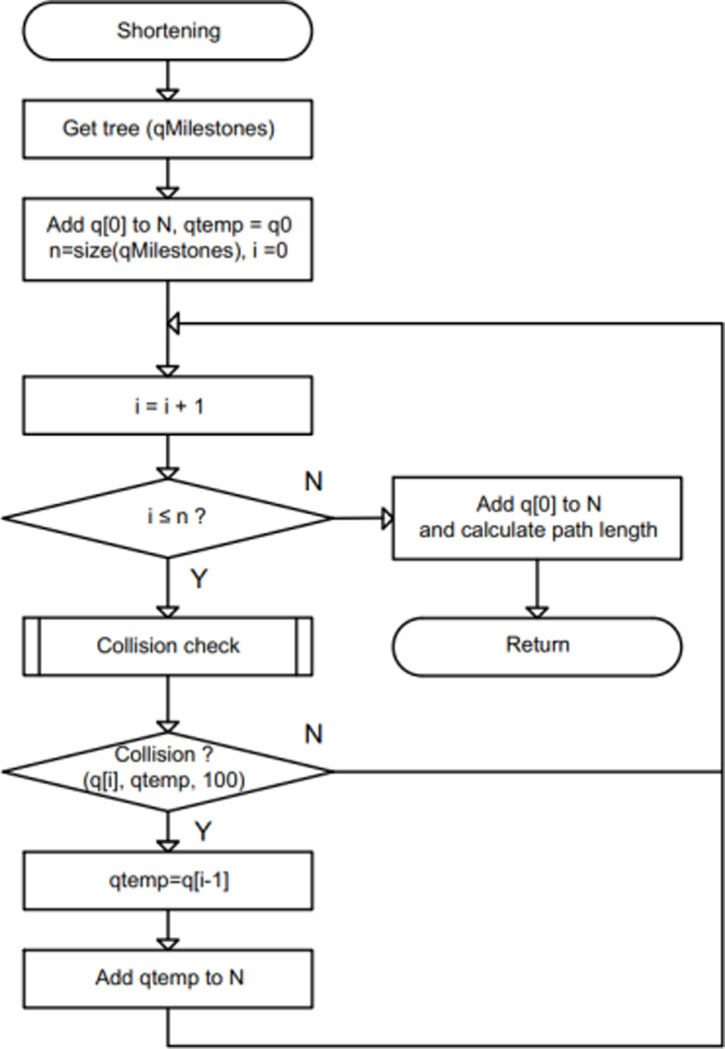
Description of traditional RRT path (red color) and post processed RRT path (green color) (a) and path shortening technique (b).

To ensure the smoothness of traveling path, cubic spline interpolation should be deployed. There are two reasons such that the spline curve produces the continuation of the acceleration vector and preserves the sufficient smoothness even in the presence of small curvatures. In Fig 14, this technique is implemented into RRT-CS scheme so that (i) the number of nodes is two, it would process linear interpolation for simplicity, and (ii) node number is larger than two, it fits several cubic spline interpolation curves through those nodes with the same pre-defined traveling time.

## 5 Simulations and experiments

**Fig 15:**
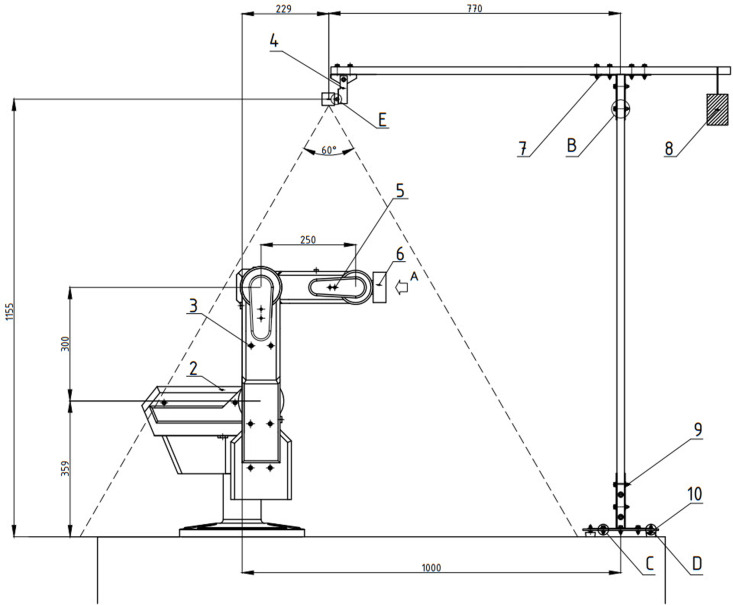
Layout setup of overall model.

**Fig 16:**
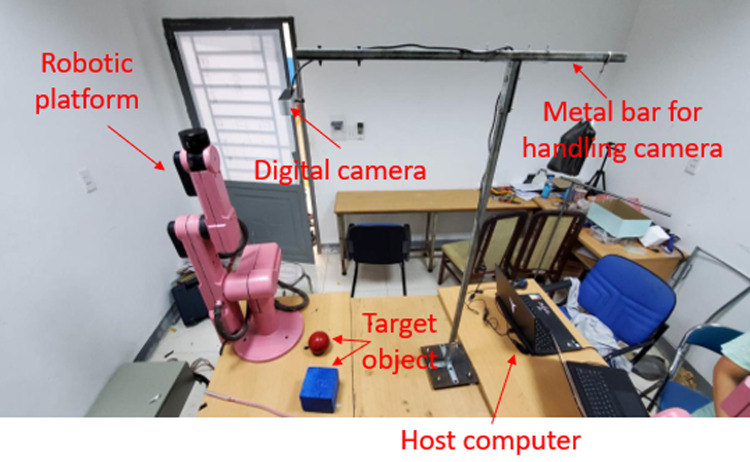
Layout setup of experimental scenario.

To prove the effectiveness and feasibility of our approach, some test scenarios have been launched as Fig 15. In front of robotic manipulator, there is a bar console to hang one digital camera. The advantages of eye-to-hand camera are to deliver global coverage, stable image and not hinder the motion of robot. Both simulation and experiment as Fig 16 are entirely executed on computer with 4.5GHz CPU Intel i7-6700HQ, 16GB RAM, card GPU NVIDIA GTX960M and Windows OS 11.

**Fig 17:**
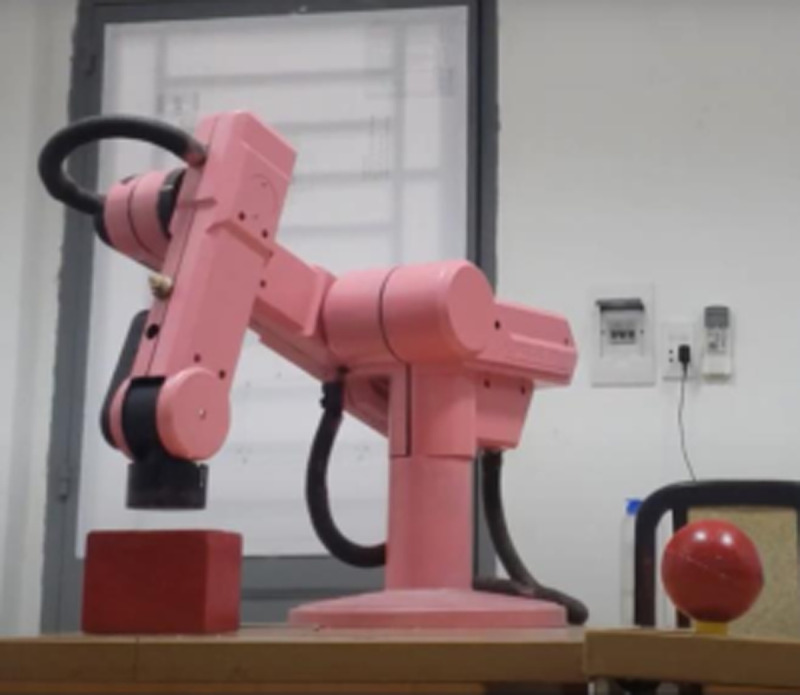
Description of the first test scenario.

**Fig 18:**
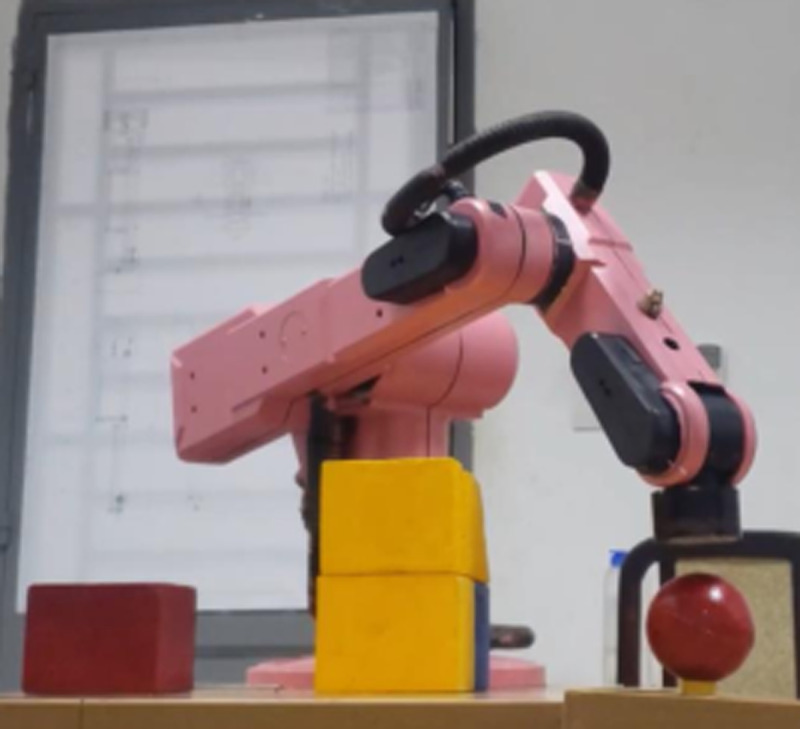
Description of the second test scenario.

**Fig 19:**
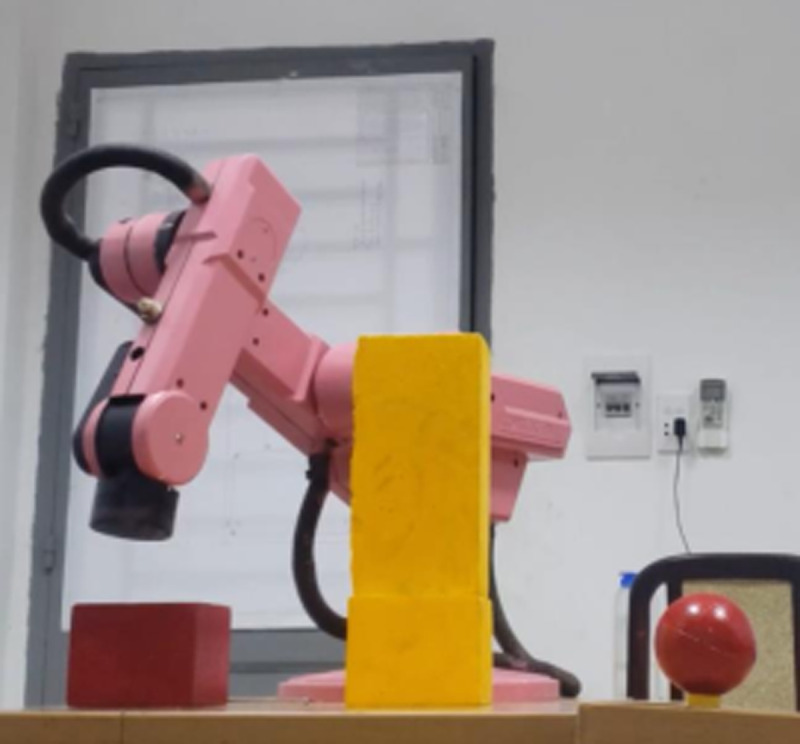
Description of the third test scenario.

Our test scene is inspired by the real-world circumstances, i.e., unknown environment which robot was not taught before. Additionally, the experimental validation of this robotic platform is proceeded in both daylight and night. In these tests, three different scenarios from free-obstacle to hard obstacle are illustrated. The first case as Fig 17 is the simplest one when manipulator only generates motion trajectory from red cube to red sphere, and there is no obstacle. In the second scenario as Fig 18, two yellow rectangular boxes are placed between cube and sphere. Our robotic manipulator must compute more flexible traveling path to avoid yellow boxes. In last case as Fig 19, the number of obstacles is more and its height is greater. To overcome these yellow boxes, robot must try its best to travel smoothly and stably.

In our research, most of validations should be evaluated by three criteria such as completeness, path quality and timing characteristics. In the view of completeness, the success rate is to identify the ability of searching the superior result if possible. Otherwise, it returns failure in finite time. Consequently, the second criterion measures path length and smoothness of trajectory. In addition, traveling time is also important to estimate how good planner is. This criterion is to assess whether motion planner is proper in the real-time applications. An offline planner would capture the environmental situation once, then it generates a path in advance. During the motion execution, it does not need to re-generate traveling trajectory until it ends. Reversely, a real-time planner must update the system state continuously and produce an up-to-date trajectory with very short time. It does not require the intervention of an operator. In the dynamic environment, this criterion becomes a very critical factor for the autonomous system.

In the sampling-based planner, the completeness of path planning means the probability of searching convergence to 1 when the number of iterations tends to infinity. For our works, each test case is validated during 50 times and comparative results are shown as Table 5. Test case 3 has the lowest number of successes runs because more obstacles are added. The first test case is perfect to plan traveling route without any obstacle. In test case 2, not all runs found a good solution and robot still makes an effort to drive.

**Table 5:** Results of evaluation in completeness.

Item	Case 1	Case 2	Case 3
*Average of iterations*	131	1066	1241
*Number of success runs/Total runs*	50/50	43/50	35/50
*Average of success rate*	100%	86%	70%

**Fig 20:**
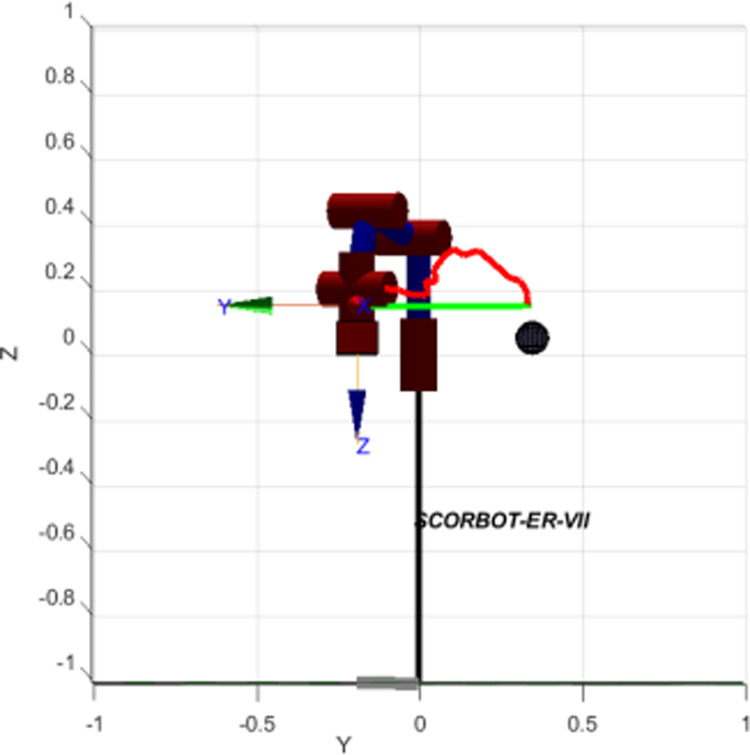
Simulation result of the proposed system using our motion planning scheme in case 1.

**Fig 21:**
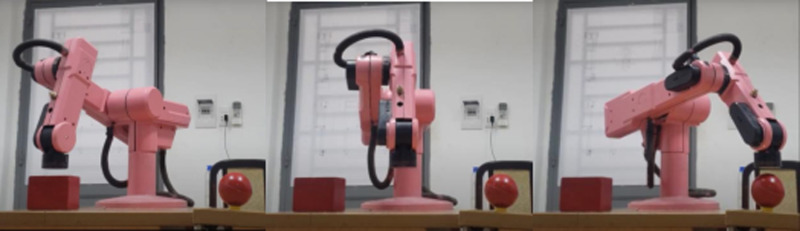
Experimental result of the proposed system using our motion planning scheme in case 1.

**Fig 22:**
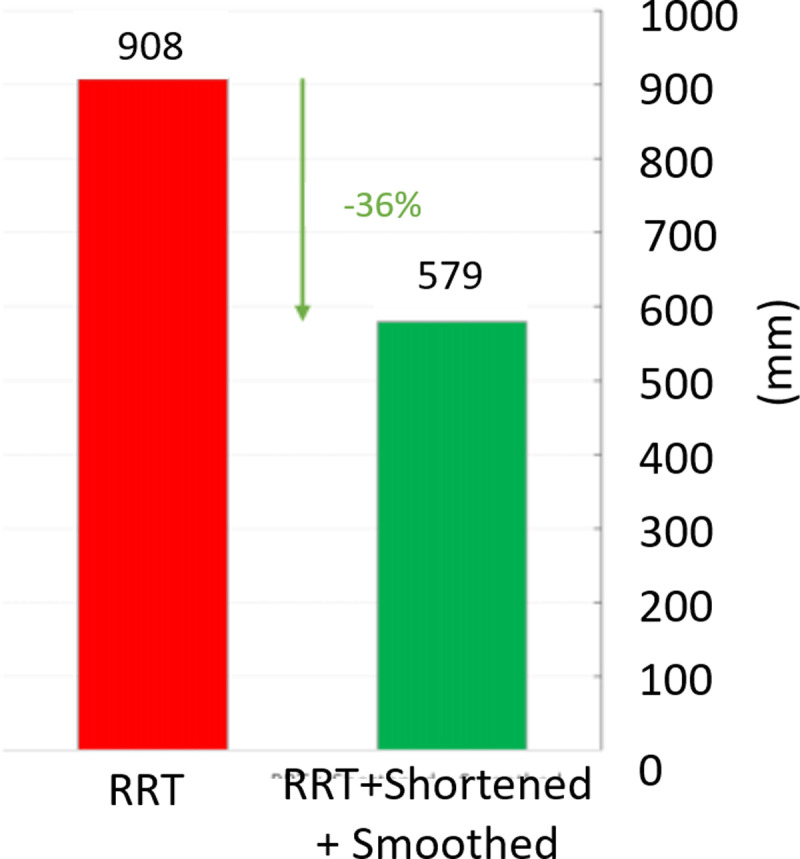
Comparative result of path length between our approach (green) and traditional RRT scheme (red) in case 1.

**Fig 23:**
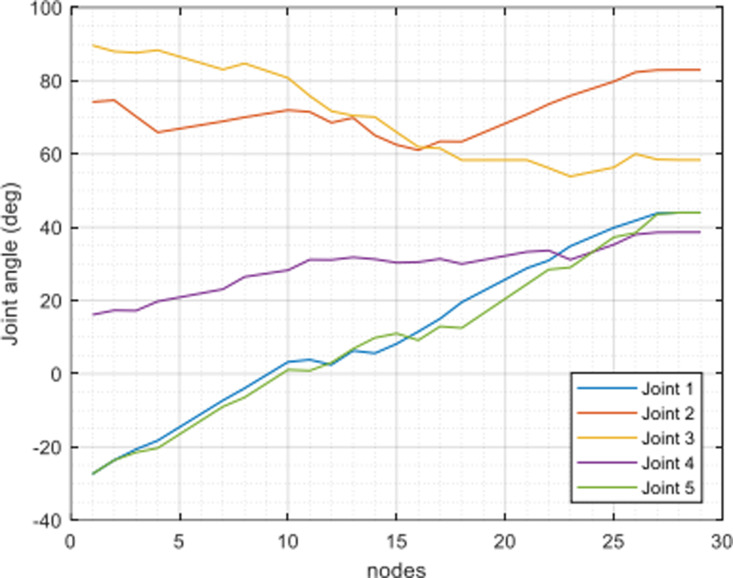
Experimental result of joint angle using traditional RRT scheme in case 1.

**Fig 24:**
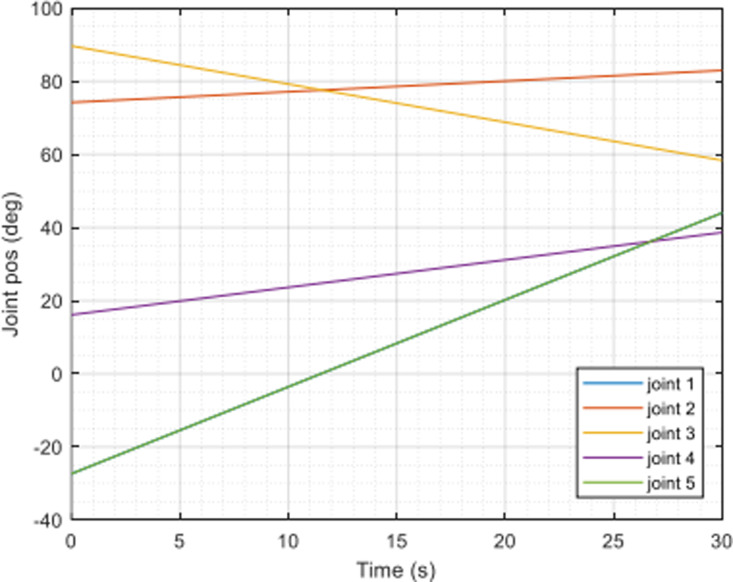
Experimental result of joint angle using our approach in case 1.

In Fig 20, path planning result by traditional RRT (red color) is still not optimal because of the random characteristics of motion planner. After using our method, traveling trajectory (green color) is re-constructed and satisfies our requirements. Fig 21 demonstrates the experimental result when our robotic hardware is used. For more details, traveling distance as Fig 22 by the proposed approach is shorter than traditional RRT, approximately 36%. Furthermore, it can be seen visually that in the test case 1, our algorithm releases the smoother curve for each joint as Fig 23 and Fig 24. These results might help to reduce any vibration in the robotic mechanism when it moves rapidly.

**Fig 25:**
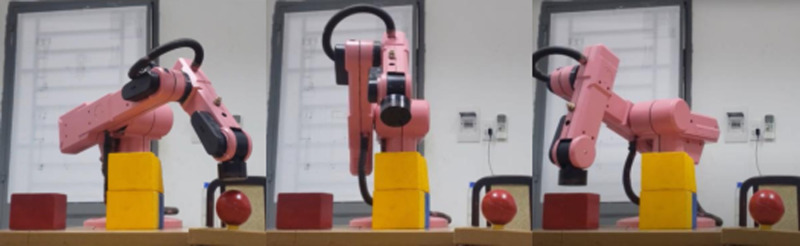
Experimental result of the proposed system using our motion planning scheme in case 2.

**Fig 26:**
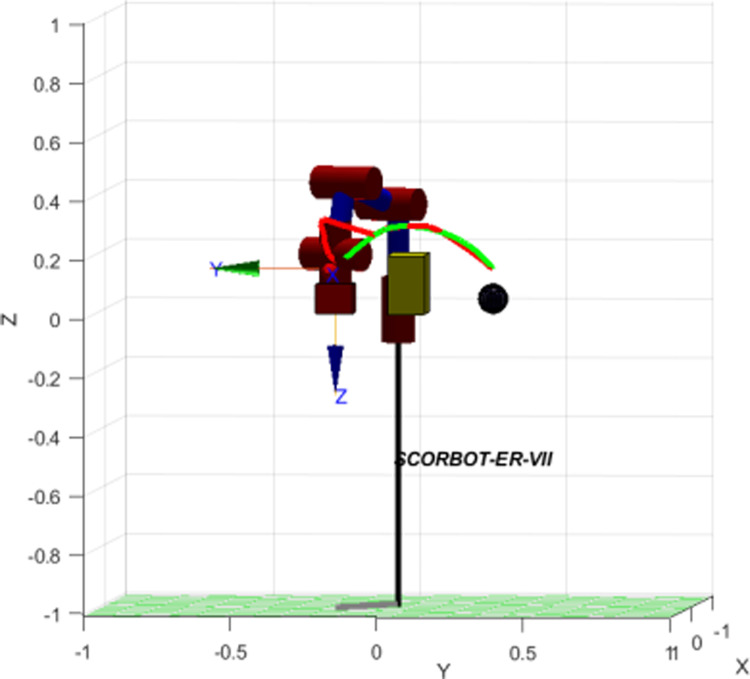
Simulation result of the proposed system using our motion planning scheme in case 2.

**Fig 27:**
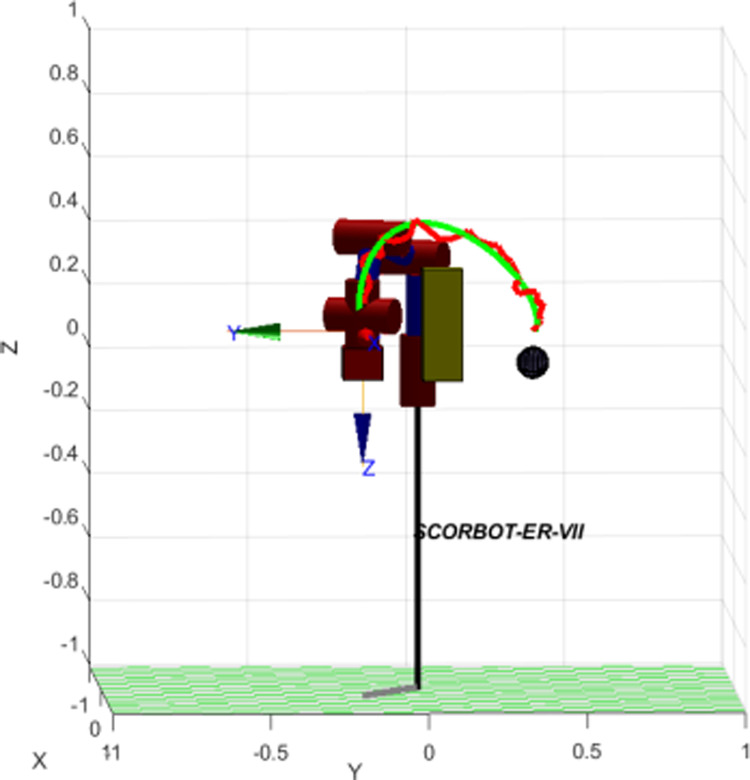
Simulation result of the proposed system using our motion planning scheme in case 3.

**Fig 28:**
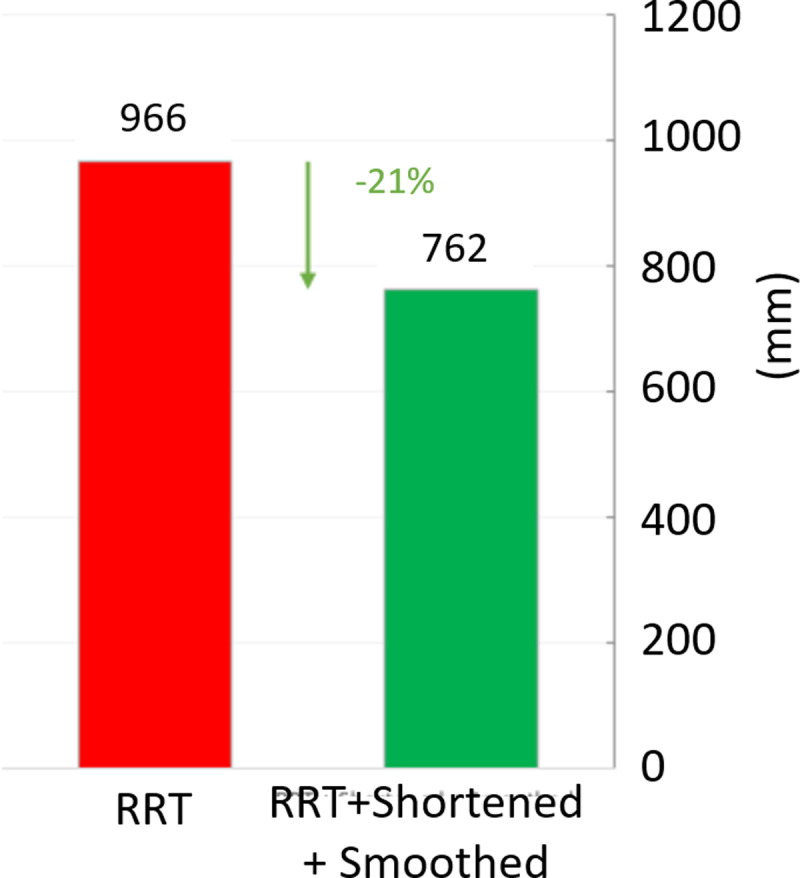
Comparative result of path length between our approach (green) and traditional RRT scheme (red) in case 2.

**Fig 29:**
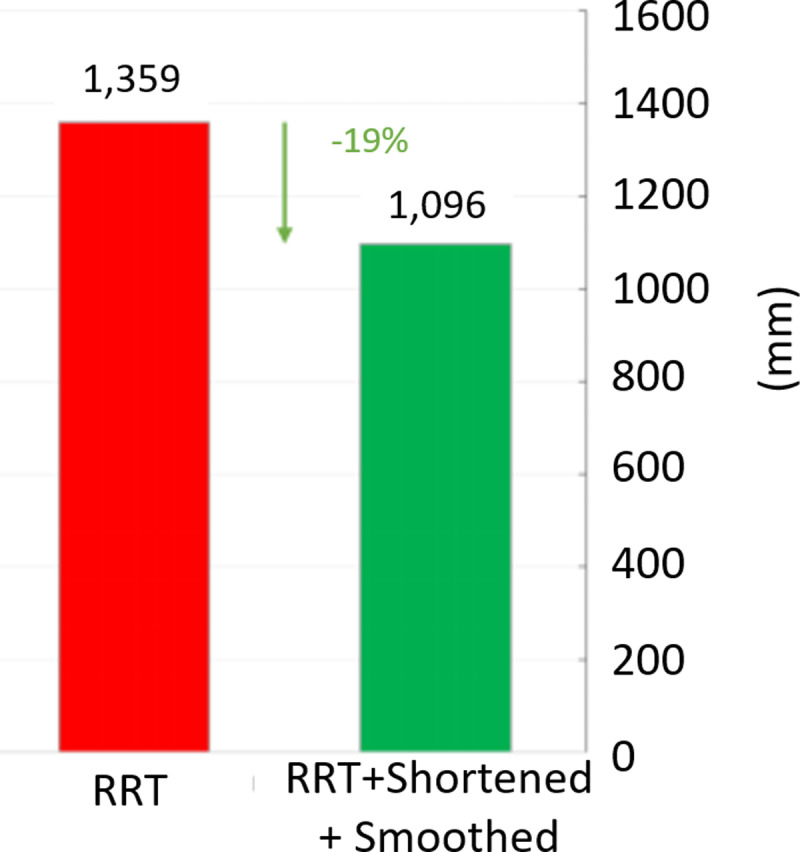
Comparative result of path length between our approach (green) and traditional RRT scheme (red) in case 3.

**Fig 30:**
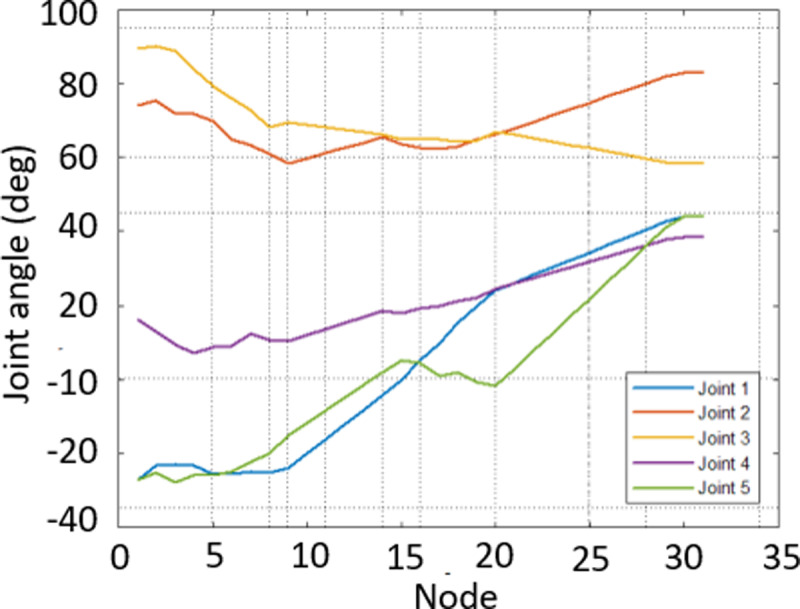
Experimental result of joint angle using traditional RRT scheme in case 2.

**Fig 31:**
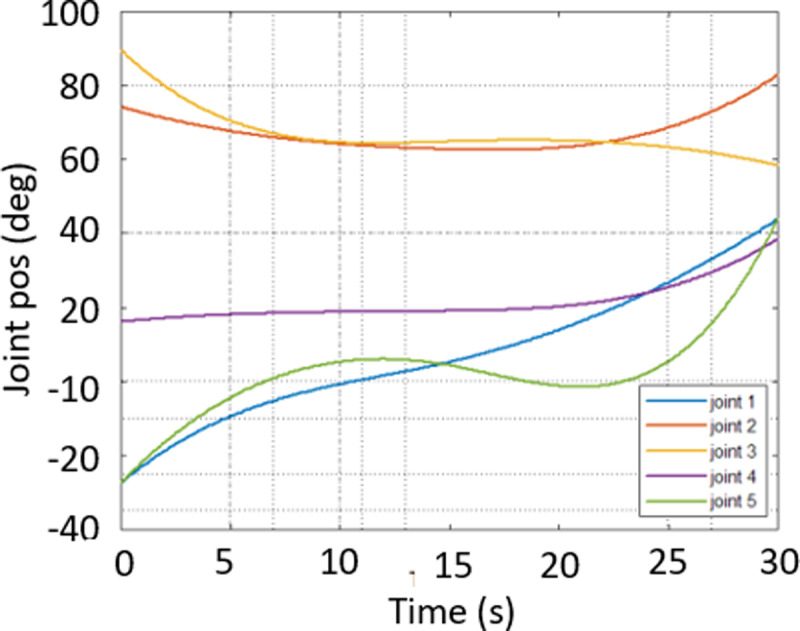
Experimental result of joint angle using our approach in case 2.

**Fig 32:**
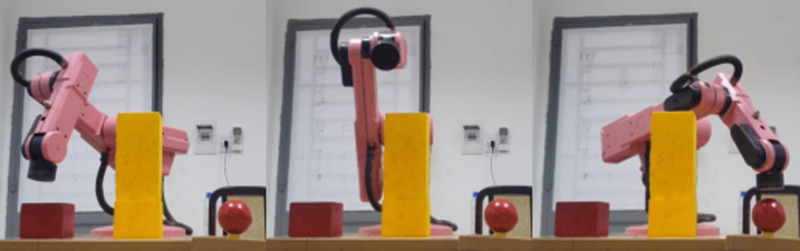
Experimental result of the proposed system using our motion planning scheme in case 3.

**Fig 33:**
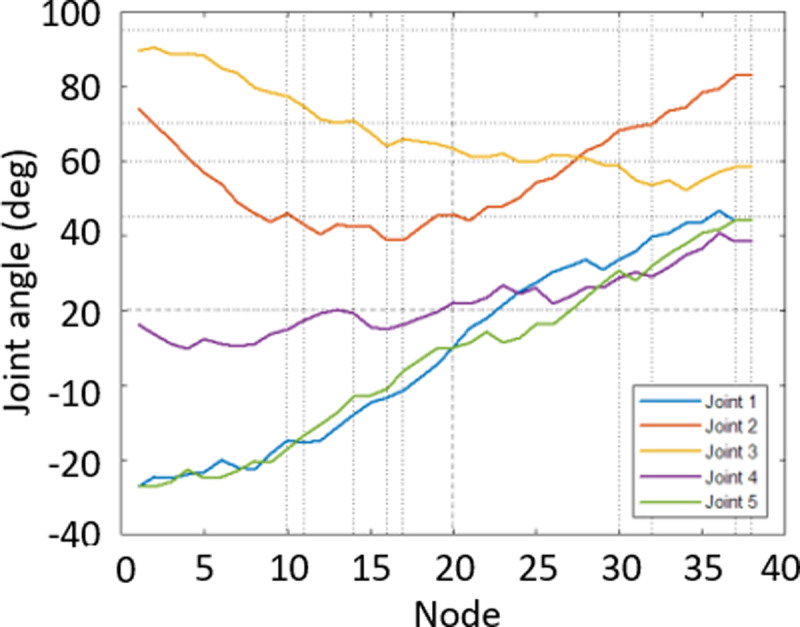
Experimental result of joint angle using traditional RRT scheme in case 3.

**Fig 34:**
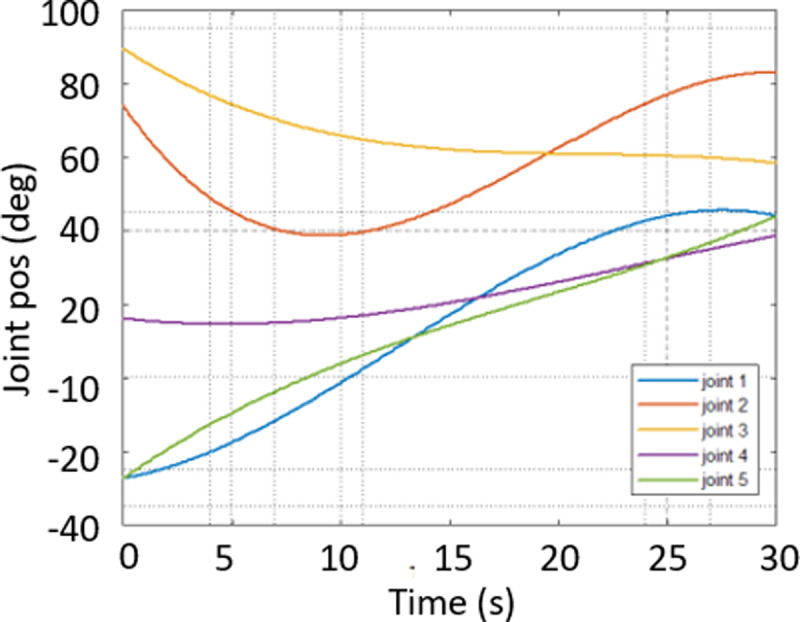
Experimental result of joint angle using our approach in case 3.

Similarly, test case 2 as Fig 25 seems to be more difficult because more obstacles are added. Theoretically speaking as Fig 26 and Fig 27, our method generates better traveling route and ensure collision-free motion. Comparing to the other method, the proposed approach achieves shorter path, roughly 21% as Fig 28 and Fig 29. It also means that robot can reach to destination in the earlier time. Besides, path planning by our scheme as Fig 30 and Fig 31 certifies the smoother curves although there exist more fluctuations owing to complex obstacles. Test case 3 as Fig 32 indicates the most complicated challenge for the reason that there are three obstacles between start and target point. Consequently, robot must try its best to overcome these obstacles. In Fig 33, host computer handles larger computations to generate more flexible paths. In our comparison, the proposed approach attains 19% shorter traveling route than traditional method. Likewise, the smoothness of motion profile as Fig 34 is reserved while robot warrants free-collision trajectory.

Table 6 describes the comparative time between different schemes. From test case 1 to test case 3, total planning time is increased proportionally in respect to the hardness of trial scenarios. In three schemes, smoothness takes the least time, i.e., less than 10%, and can be ignored if necessary. Shortening procedure spends one third portion of total processing time while RRT profile generation costs two third.

**Table 6:** List of timing comparison in various schemes.

Item	Case 1		Case 2			Case 3	
	Time (s)	Ratio (%)	Time (s)	Ratio (%)	Time (s)		Ratio (%)
*Collision valid*	0,428	34,2	0,512	33,2	0,891		46,9
*RRT*	0,820	65,6	0,925	60,0	1,135		59,7
*Shortening*	0,408	32,6	0,520	33,7	0,701		36,9
*Smoothing*	0,022	1,8	0,097	6,3	0,065		3,4
*Total*	1,250	100	1,542	100	1,901		100

Collision check time includes both collisions check in RRT and shortening algorithm.

Besides, it is essential to compute the quantitative smoothness metrics to provide a rigorous assessment as Table 7. The first indicator is path curvature (C) which measures the deviation of the trajectory from a straight-line path. If a path is perfectly smooth, the change in angle (*θ*) between consecutive waypoints should be minimal.

**Table 7:** List of timing comparison in various schemes.

Item	Traditional RRT	Proposed approach	Improvement
Path Curvature (C)	1,24	0,65	47.6% Smoother
Jerk (J)	0,93	0,52	44.1% Less Jerk
TVJA	5,87	3,21	45.3% Reduction


$$\begin{array}{c} C=\frac{1}{N}\sum {\mathrm{\backslash nolimits}}_{i=1}^{N-1}\left|\theta_{i+1}-\theta_{i}\right| \end{array}$$ (39)

where

N: Total number of waypoints in the trajectory

$\theta_{i}$: Heading angle of the robot at waypoint $i^{th}$

From equation (39), it is well-recognized that higher values of (C) would indicate more abrupt turns and less smooth motion. If C=0, the path is a perfect straight line. If (C) is large, the trajectory has many sharp turns, signifying jerky motion. Secondly, jerk (J) indicator is defined as the rate of change of acceleration. It represents how quickly acceleration varies.


$$J=\frac{1}{T}\sum_{i=1}^{N-1}\left|\frac{d^{3}x}{{dt}^{3}}\right|$$ (40)

where

T: Total time duration of motion

x: Position of the robot along the trajectory

Jerk indicator illustrates motion stability. If its value is high, the robot accelerates or decelerates abruptly. At that moment, vibrations could occur in this system. Third indicator is total variation of joint angles (TVJA).


$$TVJA=\sum_{j=1}^{M}\sum_{i=1}^{N-1}\left|q_{i+1}^{j}-q_{i}^{j}\right|$$ (41)

where

M: Number of robot joints

$q_{i}^{j}$: Joint angle of $j^{th}$ at waypoint $i^{th}$

In equation (41), it measures total changes in joint angles over the trajectory. In the case that high TVJA means joints move erratically, leading to unstable motion. Otherwise, low value of TVJA indicator specifies joint movements are gradual and smooth. In this study, we have incorporated a comparative quantitative analysis of the conventional RRT [45] and our approach. It could be observed evidently that the proposed strategy gains the improvements in both indicators, and our smoothness evaluation is quantitative, statistically valid, and technically robust.

From those results, it can be seen clearly that our technique enables rapid obstacle detection, adaptive path planning, and trajectory re-generation in dynamic environments. This integration involving RRT scheme and visual servoing technology enhances the robustness and adaptability of robotic motion planning. With the visual approach, it plays a role as the senses of the system, continuously capturing and interpreting data from surrounding. In Table 8, several competitions between our approach and conventional method are carried out.

**Table 8:** Competitive performance between traditional method and our approach.

Traditional method	Our approach
Assumes a static environment	Continuously updates the environment model in real time
Pre-planned path with no reactivity	Dynamically replans and adjusts the path on-the-fly
Slow convergence due to random sampling	Goal bias factor & adaptive sampling speed up convergence
Sharp turns and unsmoothed paths	Spline interpolation ensures smooth trajectories
Collision handling requires full re-planning	Rewiring technique reduces computation time

### Threats to validity.

Although the capsule-like model is effective for collision avoidance by reason of its smooth shape and simplified mathematical representation. Nevertheless, several barriers in handling irregular obstacles and highly dynamic environments could affect on the accurate manipulation and computational efficiency. To summarize the pros and cons of capsule-like model, Table 9 demonstrates the evaluation of possible solutions if our method is utilized. In fact, in the working environment of extremely irregular obstacles, the proposed approach could be implemented the switching mechanism to deploy capsule-like model for simplified objects and complex convex hulls or voxel-based prototype for irregular objects. Secondly, in the narrow space, capsule-based model produces a uniform safety margin which is not proper to varying constraints. An adaptive capsule of sizing design might be suitable for dense obstacles. In the highly dynamic environments, frequent re-computations of capsule-based distance could be computationally expensive. It requires to implement predictive motion model, i.e., Kalman filter or deep learning-based technique to anticipate dynamic object movements and reduce unnecessary re-computation.

**Table 9:** List of the performance validation for our model and possible solutions in the complicated environments.

Scenario	Capsule-like model	Alternative method
Simple static obstacles	Yes	Not Applied
Highly irregular obstacles	No	Convex hulls, voxel grids
Narrow corridors or gaps	Partially	Adaptive capsule radius
Fast-moving obstacles	No	Motion prediction

In point of fact, the path shortening and smoothing technique introduce additional computational overhead, but the impact depends on the implementation strategy. Path shortening removes unnecessary nodes from the RRT-generated trajectory by connecting non-consecutive nodes directly while ensuring collision-free movement. Henceforth, path shortening slightly rises computation time but remains feasible with optimizations. In term of path smoothing, it could be performed by using the flexible interpolators such as cubic spline, to generate continuous and differentiable motion trajectories. Therefore, path smoothing is computationally lightweight in most cases and feasible with local fitting methods.

## 6 Conclusions

In this study, a novel motion planner by using vision-based approach for capsule-like SCORBOT in different scenarios was developed. Primarily, robotic theory including forward kinematic and inverse kinematic, camera installation and system setup were mentioned. Several image processing techniques have been integrated in order to obtain the object coordinate, object recognition and classification. Our concept is to propose the motion planning for capsule-like model, combine the shorten and smoothen profile, as well as vision-based approach. To verify the effectiveness of the proposed method, both simulations and experiments have been accomplished. From these results, it could be seen obviously that our approach is robust, available, and feasible for the real-world applications.

Future work is a must. Our method is available for various industrial manipulators with the intricate grippers. However, with the untrained objects for instance irregular shapes or unknown profiles, it is essential to integrate the advanced machine learning techniques such as deep reinforcement learning or Q-learning for enhanced object shape recognition in cluttered environment. Besides, the adaptive control algorithms, i.e., model predictive control or adaptive neural controller, could be implemented to dynamically tune planning parameters to deal with varying constraints. In addition, collaborative multi-robot system may be explored to extend the applicability of our approach. Also, extensive simulation results with supplementary runs per scenario as well as wider experiments are needed to statistically validate robustness.

## Supporting information

S1 File Related data for this research. (RAR)
